# [^11^C]SCH23390 binding to the D_1_-dopamine receptor in the human brain—a comparison of manual and automated methods for image analysis

**DOI:** 10.1186/s13550-018-0416-2

**Published:** 2018-08-02

**Authors:** Per Stenkrona, Granville J. Matheson, Simon Cervenka, Pontus Plavén Sigray, Christer Halldin, Lars Farde

**Affiliations:** 1 Department of Clinical Neuroscience, Centre for Psychiatry Research, Karolinska University Hospital, Karolinska Institutet, R5:02, S-171 76 Stockholm, Sweden; 2 Stockholm County Council, Stockholm, Sweden; 3 PET Science Centre, Precision Medicine and Genomics, IMED Biotech Unit, AstraZeneca, Karolinska Institutet, Stockholm, Sweden

**Keywords:** Positron emission tomography, ROI, Realignment, FreeSurfer, D_1_-dopamine receptor

## Abstract

**Background:**

The D_1_-dopamine receptor radioligand [^11^C]SCH23390 has been frequently used in PET studies. In drug-naïve patients with schizophrenia, the findings have been inconsistent, with decreases, increases, and no change in the frontal cortex D_1_-dopamine receptors. While these discrepancies are likely primarily due to a lack of statistical power in these studies, we speculated that an additional explanation may be the differences due to methods of image analysis between studies, affecting reliability as well as bias between groups.

**Methods:**

Fifteen healthy subjects underwent two PET measurements with [^11^C]SCH23390 on the same day. The binding potential (BP_ND_) was compared using a 95% confidence interval following manual and automated delineation of a region of interest (ROI) as well as with and without frame-by-frame realignment.

**Results:**

Automated target region delineation produced lower BP_ND_ values, while automated delineation of the reference region yielded higher BP_ND_ values. However, no significant differences were observed for repeatability using automated and manual delineation methods. Frame-by-frame realignment generated higher BP_ND_ values and improved repeatability.

**Conclusions:**

The results suggest that the choice of ROI delineation method is not an important factor for reliability, whereas the improved results following movement correction confirm its importance in PET image analysis. Realignment is therefore especially important for measurements in patient populations such as schizophrenia or Parkinson’s disease, where motion artifacts may be more prevalent.

**Electronic supplementary material:**

The online version of this article (10.1186/s13550-018-0416-2) contains supplementary material, which is available to authorized users.

## Background

The development of molecular imaging in the 1980s was largely driven by the need to examine the dopamine system in CNS disorders such as schizophrenia and Parkinson’s disease [1, 2]. For this reason, radioligands were developed for the D_1_-dopamine receptor (D_1_-DR), the D_2_-dopamine receptor (D_2_-DR), and for the presynaptic synthesis of dopamine. In the research on the pathophysiology of schizophrenia, a large number of PET studies on the dopamine system have since then been reported and covered in reviews and meta-analyses [3, 4]. However, for the dopamine receptors and transporters, the results have been inconsistent in several respects. One example is D_1_-DR binding, which has been reported as lower, unchanged, and higher in the frontal cortex [5–10]. Medication effects may explain some of the differences between the results since experimental studies have shown that D_1_-R expression is affected by antipsychotic medication [11–13]. However, studies in drug-naïve patients have also been inconclusive [5–8]. For these analyses, sample sizes have typically been small, leading to an increased risk for both type I and type II errors [9, 13, 14]. A wider consideration is that the series of D_1_-DR studies in schizophrenia has been reported over many years during which time the methods for image analysis have been improved, including the development of new software and strategies for the definition of regions of interest (ROI) and methods for motion correction. Hence, it cannot be excluded that differences in methodological reliability may also contribute to the discrepant findings to some degree.

In the early years of PET imaging, the delineation of ROIs was performed manually directly on the PET images and guided only by the regional distribution of radioactivity. In the late 1980s, the advent of magnetic resonance imaging (MRI) allowed for manual ROI definition on individual MR images and co-registration to the PET images. Subsequently, structural brain atlases and software tools were developed for automated definition of ROIs, offering advantages in terms of reduced investigator bias and workload [15].

The agreement between manual and automated ROI delineations on PET-measured receptor-binding measurements using PET has been addressed in several studies. Good agreement has been demonstrated for tracers with widespread homogenous cortical binding [16–19] as well as more restricted subcortical binding [20, 21]. For [^11^C] raclopride binding to the D_2_-DR, which is restricted to the striatum, there was also a good agreement in repeatability as assessed using a test-retest design [21]. In contrast to the D_2_-DR, the D_1_-DR is widely expressed in the cortical regions. Importantly, the proximity of the cortical regions to the subarachnoidal space provides specific challenges for ROI definition and may also render measurements more susceptible to motion artifacts.

The primary aim of the present PET study with [^11^C]SCH23390 in healthy subjects was to compare manual and automated methods for ROI definition of both subcortical and cortical regions in a test-retest design. The secondary aim was to evaluate the effect of motion correction, and the third was to compare the use of manual and automated ROIs for the reference region, cerebellum. The methods were compared with regard to the size and spatial agreement of the ROIs, the binding potential (BP_ND_) values, and repeatability of the BP_ND_ values.

## Methods

Sixteen healthy subjects, age 22–35 years, were enrolled at the Center for Psychiatry Research, Department of Clinical Neuroscience, Karolinska Institutet, and Stockholm County Council, Stockholm, Sweden. One subject was excluded from the analysis due to incorrect head positioning in the PET system rendering a small portion of the cerebellum visible in only one image section. All subjects were male since this study was made in preparation for a planned phase 1 trial on drug-induced D_1_-DR occupancy.

The subjects were healthy according to medical history, physical examination, MRI, blood and urine chemistry, and psychiatric screening based on M.I.N.I. interview, Becks Anxiety Inventory, the Montgomery-Åsberg Depression Rating Scale, and the Alcohol Use Disorders Identification Test (AUDIT). They had no history of alcohol or drug addiction or abuse [22], frequent nicotine use, or history or presence of epilepsy and brain injury. A urine drug screening was performed at screening and before each PET measurement.

All subjects underwent MRI, performed on a 1.5-T Siemens Avanto imaging system (Siemens AG, Muenchen, Germany) at Praktikertjänst Röntgen, Odenplan, Stockholm, Sweden. Each individual underwent one MR examination. A T2-weighted measurement was performed to rule out any brain abnormality, and a T1-weighted measurement was performed with isometric 1-mm voxels used for gray and white matter segmentation and delineation of ROIs. The T2 protocol used the following sequence: repetition time/echo time = 4990/100 ms, field of view 230 mm, image matrix 18 blades, flip angle 150°, and slice thickness = 5 mm. The T1 protocol used a 3D sagittal magnetization-prepared rapid gradient-echo (3D MP-RAGE) with the following sequence: repetition time/echo time/inversion time = 1790/3.53/1100 ms, field of view 260 mm, image matrix 256 mm × 208 mm, flip angle 15°, and slice thickness = 1 mm.

For each subject, examinations were performed twice on the ECAT EXACT HR PET system, in the morning and afternoon, respectively. Radioactivity in the brain was measured with 3D data acquisition. The spatial resolution in the reconstructed sections is 3.6 mm at the center of the field of view [23]. A transmission scan was performed using three rotating ^68^Ge rod sources for 5 min.

To minimize head movement during the PET measurement, a plaster helmet was made for each subject individually, for use during the PET measurement [24]. At the start of the PET measurement, a sterile phosphate buffer (pH = 7.4) containing [^11^C]SCH23390 was injected as a bolus during several seconds into the cubital vein. The venous catheter was then immediately flushed with up to 10 mL saline solution.

The injected radioactivity was 333 ± 44 and 332 ± 49 MBq (mean, SD), in the morning and the afternoon, respectively, with a specific radioactivity of 507 ± 244 and 574 ± 238 MBq/nmol, which correspond to a mass of 0.23 ± 0.13 μg and 0.20 ± 0.10 μg (mean ± SD) [25].

Following injection, emission data were collected for 51 min in a sequence of 13 time frames. The time frames of acquisition data were reconstructed into a series of 3D PET images of radioactivity concentration. Images were reconstructed to correct for attenuation and scatter using 2D filtered back projection, with a Hanning filter (2.0 mm) on a 128 × 128 matrix and azoom = 2.17. The voxel size was 2.030 × 2.030 × 3.125 mm.

### MR and PET image processing

#### Processing of MRI and PET data

The PET images were corrected for head movement using a frame-by-frame realignment algorithm, in which all frames were individually realigned to the first minute of acquisition [26] using SPM5 (Wellcome Department of Cognitive Neurology, University College London) [27]. For comparison, a parallel data set was created for which no realignment was performed.

The T1-weighted MR images were reoriented according to the line defined by the anterior and posterior commissures (AC-PC line) being parallel to the horizontal plane and the inter-hemispheric plane parallel to the sagittal plane. The MR images were then co-registered to the summation PET image (9–51 min) using SPM5 (Wellcome Department of Imaging Neuroscience, London, UK) using the normalized mutual information algorithm [28] and the default 7 × 7 FWHM smoothing of the 256 × 256 joint histogram.

The MR images were used to delineate anatomical regions of interest (ROI) for the caudate nucleus (CAU), the putamen (PUT), the dorsolateral prefrontal cortex (DLPFC), and the insular cortex (INS). Each region was defined by a manual and an automated method, respectively. The regions were chosen to represent the regions of central interest in schizophrenia research and include high- and low D_1_-DR density regions with different degrees of proximity to CSF. Regions with more CSF borders may be particularly vulnerable to errors in ROI definition or motion artifacts. The caudate and putamen are high-D_1_-DR density regions bordering and not bordering to CSF, respectively. The DLPFC and INS are low-density D_1_-DR regions bordering more and less to CSF, respectively.

#### Manual ROIs

For the manual method, an in-house software, HBA [29], was used where the reoriented MP-RAGE volume was loaded for manual delineation of the ROIs on any of the three orthogonal projections. The manual segmentation was performed by one investigator (PS) who has more than 20 years of experience in manual ROI delineation. The CAU and PUT were delineated as described by Mawlawi et al. [30], with the modification that the sagittal planes were used instead of the coronal. The DLPFC was traced on all the coronal planes anterior to the genu of the corpus callosum. The INS was delineated on all of its transaxial planes. The cerebellum was drawn on the central six transaxial images of the cerebellum separately on each hemisphere and about 1 cm distant from the subarachnoidal space. The manual ROIs were not masked by the GM map in order to have a complete manual method of gray-white matter segmentation for comparison with the automated ROIs that were masked by the GM map. The ROIs were translated into each PET study space using the inter-modality co-registration matrices.

#### Automated ROIs

The automated definition of target ROIs was performed using FreeSurfer (FS, version 5.0.0, http://surfer.nmr.mgh.harvard.edu/) [31] to obtain subject-specific anatomical delineation by reconstruction of the cortex and segmentation of subcortical structures as described elsewhere [32, 33]. The FreeSurfer morphometric procedures have been shown to exhibit good reproducibility across scanner manufacturers and across different field strengths [34, 35] and have been validated against histological [36] and manual measurements [37]. In addition, the cortical structures are divided based on individual cortical folding patterns to match the cortical geometry across subjects [38].

The ROI for the dorsolateral prefrontal cortex used here comprises the ctx-rostralmiddlefrontal and ctx-parstriangularis regions of the Desikan-Killiany Atlas in FreeSurfer [39].

Finally, an automated ROI for the cerebellum was defined using FSL (the FMRIB Software Library), a library of analysis tools for brain imaging data [40]. This region was defined using the maximum probability FSL MNIfnirt atlas segmentation with 25% probability threshold [41], from which a mask was defined containing cerebellar regions VI, crus I, and crus II (indices [5, 7, 8, 10, 11, 13]). This mask was then registered to the space of each individual’s MR using the inverse FNIRT warp parameters. This mask was subsequently then trimmed as follows: 8 mm from the cortex, 8 mm from the vermis (defined using the same atlas), 4 mm from the edge of the brain mask (FSL brain mask). In addition, voxels belonging to the two most inferior planes of the PET image were excluded from the ROI. The resulting ROI was multiplied by the FreeSurfer gray matter segmentation mask to obtain a ROI consisting of only those voxels identified as belonging to gray matter. For more information, see Matheson et al. [42]. The purpose was to compare the different impact on the BP_ND_ value using a standardized automatically delineated cerebellum compared to using the manual cerebellum as reference region.

#### Time-activity curves

The two ROI data sets (manual and automated) were applied to extract time-activity curves (TACs) from the four dynamic PET images (test and retest each processed with or without realignment) amounting to eight sets of TACs.

### Calculation of BP_ND_ values

The regional BP_ND_ values for [^11^C]SCH23390 binding to D_1_-DR were calculated with the simplified reference tissue model using the cerebellum curve as an estimate for non-specifically bound [^11^C]SCH23390 [43]. The eight sets of TACs were analyzed twice using the manual and modified FSL cerebellum ROI-derived TAC, respectively, amounting to 16 sets of BP_ND_ values for each measurement.

### Comparison of ROI volumes

To compare manually and automatically generated ROIs (FreeSurfer and FSL), the number of voxels for each paired ROI (both hemispheres) was extracted from the ROI files and converted to cubic millimeter. The spatial agreement between the methods was expressed by the Jaccard coefficient which is the ratio between the size of the intersection and the size of the union of the voxel sets:

1 $$ J\left(A,B\right)=\frac{\left|A\cap B\right|}{\left|A\cup B\right|} $$

The coefficient can vary between 0 (no agreement) and 1 (perfect match).

In addition, the spatial agreement was estimated for the relatively smaller manual ROI by the percentage of the manual ROI covered by the FreeSurfer or FSL ROI:

2 $$ \frac{\left|A\cap B\right|}{\mathrm{Manual}\ \mathrm{ROI}\ \mathrm{volume}}\times 100 $$

The values increase with the overlap of the manual and automated ROIs so that 100% indicates that the manual ROI is completely encompassed by the FreeSurfer or FSL ROI.

### Statistics

#### Statistical analysis—general

In the following, the terminology related to the statistical analysis follows the recommendations of the Terminology Working Group of the Quantitative Imaging Biomarker Alliance [44]. The inclusion of a test-retest analysis serves two purposes. First, it allows for the examination of the repeatability of PET measurements with [^11^C]SCH23390. Second, it can be viewed as a duplicate measure of the same variable, i.e., in the same way as the mean of triplicate measurements commonly are used in biochemistry and bioanalyses.

#### Test-retest analysis

Repeatability was measured by calculating the absolute variability (AV) expressed as the difference in the BP_ND_ values between the first and second PET measurements relative to the mean of the two values according to the following equation:

3 $$ \mathrm{AV}=\frac{2\times \mid {\mathrm{BP}}_{\mathrm{ND}}^{\mathrm{PET}1}-{\mathrm{BP}}_{\mathrm{ND}}^{\mathrm{PET}2}\mid }{{\mathrm{BP}}_{\mathrm{ND}}^{\mathrm{PET}1}+{\mathrm{BP}}_{\mathrm{ND}}^{\mathrm{PET}2}} $$

The intraclass correlation coefficient (ICC) was used as a relative measure of reliability in BP_ND_, where the variability between the test and retest BP_ND_ is related to the variability of BP_ND_ among the subjects according to the following equation:

4 $$ \mathrm{ICC}=\frac{{\mathrm{MS}}_{\mathrm{B}}-{\mathrm{MS}}_{\mathrm{W}}}{{\mathrm{MS}}_{\mathrm{B}}+\left(k-1\right){\mathrm{MS}}_{\mathrm{W}}} $$

where MS_B_ is the between-subject mean sum of squares, MS_W_ is the within-subject mean sum of squares, and *k* represents the number of observations (in this case 2). The ICC is an estimation of reliability, which is the “true” variance/total variance. Thus, a value of 1 means that there is no measurement error at all, while a value of 0 means that the obtained measurements are entirely comprised of measurement error. Zero is therefore technically the lowest value for the ICC, and a negative value can be approximated by 0.

#### Comparisons of ROI volumes and BP_ND_ values between the methods for image analyses

The advantages of interval estimate compared to point estimate as the output of statistical analysis have been demonstrated for medical research in general [45, 46] and specifically for the comparison of quantitative biomarkers [47]. Hypothesis testing for a point estimate using a *p* value is a binary (yes or no) decision even for minor differences that may not be of practical importance [48]. It may thus be more useful to evaluate the data with an interval estimate that in addition to a *p* value provides a plausible range for the true value, which can be judged subjectively on an agreement between and precision of the methods [49].

Computation of median, interquartile range (25th to 75th), and coefficient of variation (CoV; $\frac{\mathrm{standard}\ \mathrm{deviation}}{\mathrm{mean}}$) was applied to evaluate the distribution and variability of the BP_ND_ values. Computation of a 95% confidence interval was applied for the evaluation of the agreement and precision of the two ROI volumes (manual and automated), and the two BP_ND_ values from the three comparisons in the image analysis (ROI definition, realignment and definition of reference region).

The two PET measurements and the two methods in each of the three comparisons of the analysis amounted to 2 × 2 × 2 × 2 equals 16 sets or 8 pairs of BP_ND_ values for each ROI as illustrated in Additional file 1: Table S1 for the test-retest combinations. The balanced data set made it possible to test for agreement between the pairs of BP_ND_ values only once instead of for each of the eight combinations of the test-retest and three pairs of methods of analysis (manual target ROI/automated target ROI, no realignment/realignment, manual reference ROI/automated cerebellum ROI). Hence, the statistical computations were applied once for each pair of BP_ND_ values using the mean BP_ND_ value of the other eight combinations of data (2 × 2 × 2). This averaging was justified after testing by ANOVA for the absence of two- and three-way interactions among the pairs of variables.

The two absolute variability (AV) values and ICC values were also evaluated for agreement and precision by the mean differences and confidence intervals in the same way as for the BP_ND_ values with the difference that there were 2 × 2 × 2 (i.e., eight sets or four pairs) AV and ICC values for each of the three parts of the image analysis. The comparison of ICC values between the methods was assessed by using the 95% confidence intervals [50] to examine whether there was an overlap between the confidence intervals between the pairs of methods compared.

## Results

The subjects completed the study according to the protocol. Each subject underwent one T1 MRI scan on 1 day and two PET measurements in the morning and afternoon of another day after a negative urine drug screen on the same day. The time gap between PET and MRI studies was 1 to 96 days, median 6 days.

The spatial agreement of the manual and FreeSurfer ROIs estimated by the Jaccard coefficient varied between 32 and 69% (Table 1). The highest values were obtained for the caudate (59%), putamen (57%), and insula (69%), indicating that the ROIs overlapped to a major extent. The percentage of the manual ROI covered by the FreeSurfer ROI varied between 51.3 and 92.5%.

**Table 1:** Jaccard coefficients obtained by comparison of ROIs made manually and by FreeSurfer

ROI	Jaccard coefficient	Intersect (voxels)	Intersect % of manual volume
CAU	0.59	450	92.5
PUT	0.57	614	92.0
DLPFC	0.32	1756	56.2
INS	0.44	652	79.9
CBL	0.32	291	51.3

The ROI volumes were calculated by the number of voxels multiplied by the voxel volume for each paired ROI (both hemispheres) extracted from the ROI files. Worth noting is that the positioning of the head in the gantry differed slightly between the two PET measurements, and the subsequent two sets of re-sliced ROIs in PET space differed in volumes by 1.7 ± 3.0% (mean ± SD).

**Fig 1:**
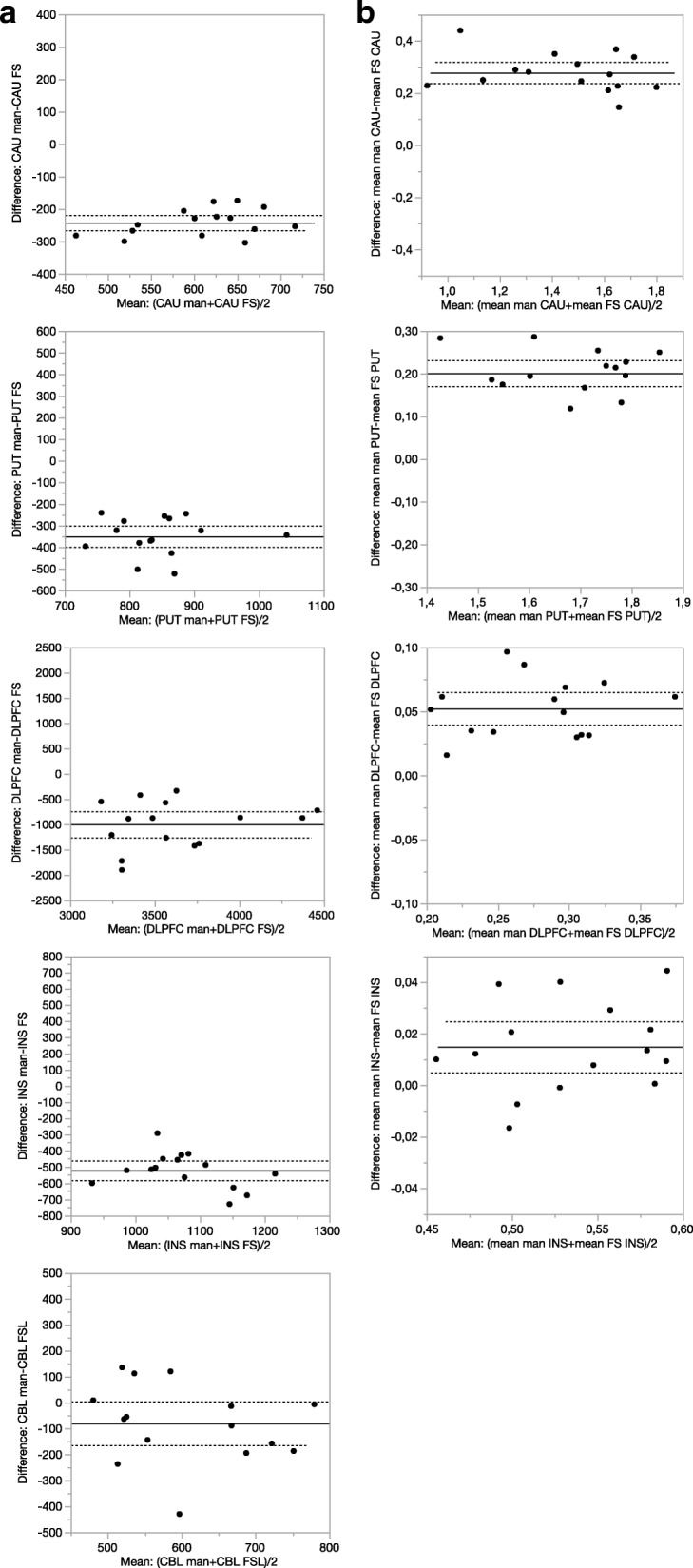
Bland-Altman plots with the mean and 95% confidence interval of ROI volumes (**a**) and binding potential (BP_ND_) of [^11^C]SCH23390 (**b**) of the caudate, putamen, dorsolateral prefrontal cortex, and insula derived from manual (man) and FreeSurfer (FS) methods. Man = manual, FS = FreeSurfer, CAU = caudate, PUT = putamen, DLPFC = dorsolateral prefrontal cortex, and INS = insula

**Fig 2:**
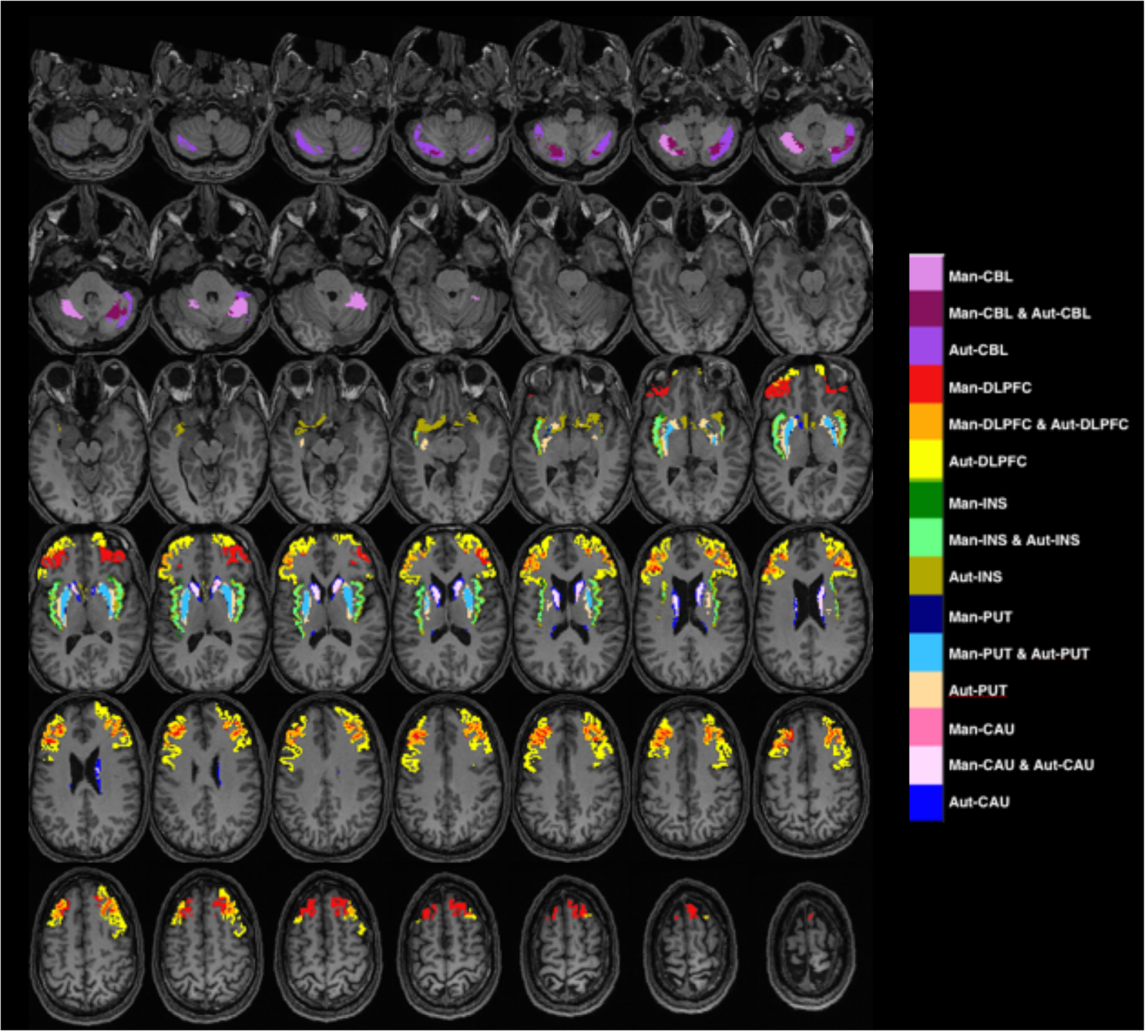
Color-coded voxels of the manual (Man) and automated (Aut) ROIs superimposed on the corresponding MR image of a healthy volunteer selected at random. ROIs generated manually are superimposed on those generated by FreeSurfer. CAU = caudate, PUT = putamen, DLPFC = dorsolateral prefrontal cortex, INS = insula, and CBL = cerebellum

Each of the FreeSurfer ROIs and the modified FSL cerebellum ROI were significantly larger compared to the corresponding manual ROIs (Figs. 1a and 2, Table 2). The 95% CI for the differences was relatively narrow indicating a high precision for both methods (Fig. 1a, Table 2). The CoV (%) for the manual ROI volume was generally higher than for the automatically generated ROIs.

**Table 2:** ROI volumes and binding potential (BP_ND_) values for [^11^C]SCH23390 binding obtained by two repeated PET measurements analyzed by three different comparisons of methods: (1) ROI definition (manual (Man)/FreeSurfer (FS)), (2) movement correction (no realignment/realignment), and (3) definition of the cerebellum as reference region (manual/modified FSL)

	CAU		PUT		DLPFC		INS		CBL	
ROI volume by ROI method (mL)	Man	FS	Man	FS	Man	FS	Man	FS	Man	FSL
Median	6.5	9.5	8.4	13.0	39.3	53.7	10.8	16.6	7.6	8.1
Interquartile range	5.7–8.8	8.8–9.9	8.0–9.4	12.4–13.8	37.4–43.4	49.2–57.2	10.0–11.1	16.4–18.2	6.3–8.3	6.9–10.1
CoV %	16.4	8.9	12.9	8.4	15.8	10.0	9.1	8.0	19.2	21.1
Mean diff (CI 95%)	− 3.1 (− 3.4, − 2.8)^a^		− 4.5 (− 5.0, −3.9)^a^		− 12 (− 15.9, − 9.8)^a^		− 6.7 (− 7.4, − 6.0)^a^		− 1.0 (− 2.0, − 0.4)^a^	
BP_ND_ by ROI method	Man	FS	Man	FS	Man	FS	Man	FS		
Median	1.65	1.39	1.85	1.62	0.32	0.26	0.55	0.53		
Interquartile range	1.43–1.76	1.14–1.52	1.72–1.88	1.49–1.68	0.26–0.33	0.21–0.29	0.50–0.58	0.50–0.57		
CoV %	16.1	20.9	7.0	8.9	17.0	19.4	8.8	8.4		
Mean diff (CI 95%)	0.28 (0.24, 0.31)^a^		0.20 (0.17, 0.23)^a^		0.05 (0.04, 0.06)		0.01 (0.01, 0.02)			
BP_ND_ by realignment	No realign	Realign	No realign	Realign	No realign	Realign	No realign	Realign		
Median	1.46	1.57	1.74	1.73	0.27	0.30	0.54	0.53		
Interquartile range	1.24–1.63	1.33–1.65	1.61–1.81	1.61–1.75	0.21–0.29	0.26–0.32	0.50–0.58	0.50–0.58		
CoV %	22.1	14.7	9.0	9.7	21.2	15.4	8.8	8.2		
Mean diff (CI 95%)	− 0.10 (− 0.16, − 0.05)^a^		0.00 (− 0.03, 0.03)		− 0.04 (− 0.05, − 0.02)^a^		0.00 (− 0.01, 0.01)			
BP_ND_ by reference region	Man CBL	FSL CBL	Man CBL	FSL CBL	Man CBL	FSL CBL	Man CBL	FSL CBL		
Median	1.49	1.53	1.70	1.77	0.28	0.29	0.52	0.55		
Interquartile range	1.26–1.62	1.31–1.67	1.58–1.76	1.63–1.80	0.22–0.30	0.25–0.31	0.49–0.57	0.51–0.58		
CoV %	18.7	17.6	8.3	7.3	18.8	16.9	8.8	8.3		
Mean diff (CI 95%)	− 0.04 (− 0.06, − 0.03)^a^		− 0.05 (− 0.06, − 0.03)^a^		− 0.02 (− 0.03, − 0.01)^a^		− 0.03 (− 0.03, − 0.01)^a^			

The values were obtained from four brain regions in 15 subjects, each value represents the mean of the eight values for that measurement or part of the analysis. Interquartile range = 25th to 75th percent

*CoV* coefficient of variation, *CI* confidence interval

^a^The confidence interval does not contain zero

**Fig 3:**
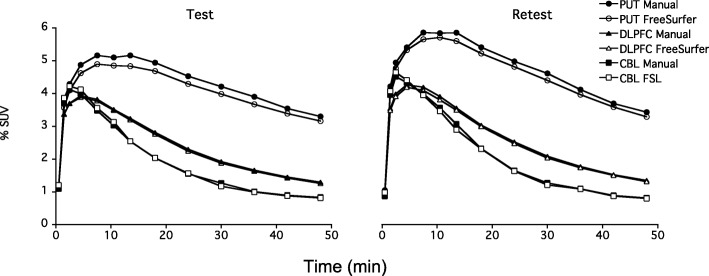
Time-activity curves from a healthy volunteer after i.v. injection of [^11^C]SCH23390 in the morning (left) and afternoon (right). The curves were derived from realigned dynamic PET images using manually and automatically generated ROIs, respectively. PUT = putamen, DLPFC = dorsolateral prefrontal cortex, CBL = cerebellum. SUV = standardized uptake value = (C_ROI_/inj dose) × body weight

As can be seen from the TACs, there was a rapid initial increase of brain radioactivity that peaked after 5–10 min followed by a rapid decline (Fig. 3). The AUC of the TACs for the realigned PET images was 2–18% higher for the manually generated target ROIs when compared to that of the FreeSurfer (*p* < 0.001; data not shown) (Fig. 3). The AUC of the cerebellum TACs were similar in the beginning and 3% higher during the last 24 min for the manual CBL compared to modified FSL CBL (*p* < 0.001 for the last 24 min; data not shown).

The eight sets of target TACs and two sets of cerebellum TACs amounted to 16 BP_ND_ values for each ROI (Additional file 1: Table S1). The statistical computations of the regional BP_ND_ values for the three parts of the analysis are listed in Table 2. The 95% CI for most of the differences was relatively narrow and did not contain zero indicating statistical significance for the difference between methods (Fig. 1b). The manually generated BP_ND_ values were 3–21% higher with a generally low CoV and interquartile range compared to that of the automatically generated BP_ND_ values. After realignment, the BP_ND_ values became higher for the caudate and DLPFC (Table 2). On the contrary, the BP_ND_ values for putamen and insula did not increase after realignment. The use of the modified FSL cerebellum ROI increased BP_ND_ by 3 to 7% in all regions as compared to that of the manual CBL.

The absolute variability values (AV) for each of the four regional BP_ND_ values were calculated for the total of eight pairs of regional BP_ND_ values from the three parts of the analysis (2 × 2 × 2 = 8) (Additional file 1: Table S1). The AV values among the methods varied between 3.9–11.9% for the caudate and putamen and 8.0–17.9% for the DLPFC and insula. The 95% CI of the mean difference in AV was wide and included zero in most cases indicating no significant differences (Table 3). However, following realignment, the 95% CI of the mean difference in AV did not overlap with zero for caudate, putamen, and DLPFC, suggesting a small but significant improvement. The use of automated cerebellum did not improve the AV values in any region compared to that of the manual. The ICCs were generally higher using FreeSurfer, but all comparisons fell within the limits of the 95% confidence intervals, meaning that these differences are not significant.

**Table 3:** Two sets of mean absolute variability (AV) values and intraclass correlation coefficients (ICC) of the binding potential (BP_ND_) of [^11^C]SCH23390 in four brain regions derived from two different methods (each defined in the leftmost column) in each of three parts of the image analysis process: (1) manual/FreeSurfer ROIs, (2) no realignment/realignment, and (3) manual cerebellum ROI/automated FSL cerebellum ROI)

Method (1/2)	ROI	AV (%) method 1	AV (%) method 2	AV diff (95% CI)	ICC (95% CI) Method 1	ICC (95% CI) Method 2
ROI definition Method 1: manual Method 2: FreeSurfer	CAU	9.45	9.79	1.61 (− 1.96, 1.27)	0.79 (0.49, 0.92)	0.87 (0.66, 0.95)
	PUT	4.82	4.72	0.95 (− 0.86, 1.05)	0.73 (0.38, 0.90)	0.84 (0.59, 0.94)
	DLPFC	13.17	14.30	2.67 (− 3.79, 1.55)	0.72 (0.36, 0.89)	0.72 (0.36, 0.90)
	INS	9.73	7.94	1.76 (0.03, 3.54)	0.31 (− 0.25, 0.70)	0.46 (− 0.07, 0.78)
Movement correction Method 1: none Method 2: realignment	CAU	13.78	6.81	4.43 (2.55, 11.40)	0.79 (0.50, 0.93)	0.86 (0.64, 0.95)
	PUT	5.88	3.76	1.68 (0.45, 3.80)	0.75 (0.40, 0.91)	0.81 (0.53, 0.93)
	DLPFC	19.48	10.08	6.08 (3.32, 15.48)	0.63 (0.21, 0.86)	0.77 (0.44, 0.91)
	INS	9.40	8.47	1.50 (− 0.56, 2.44)	0.38 (− 0.18, 0.74)	0.37 (− 0.18, 0.74)
Definition of reference region (cerebellum) Method 1: manual Method 2: FSL	CAU	10.60	9.15	1.86 (− 0.41, 3.30)	0.81 (0.53, 0.93)	0.84 (0.59, 0.94)
	PUT	5.37	4.38	2.04 (1.05, 3.03)	0.77 (0.45, 0.91)	0.76 (0.41, 0.91)
	DLPFC	15.29	11.54	6.08 (− 2.33, 9.82)	0.71 (0.33, 0.89)	0.73 (0.38, 0.90)
	INS	10.73	7.88	2.81 (0.05, 5.66)	0.23 (−0.34, 0.66)	0.50 (− 0.02, 0.80)

## Discussion

The main objective of the present study was to examine the impact of specific methodological steps used in quantitative image analysis of [^11^C]SCH23390 binding to D1-DR on variability and reliability. The methods were chosen to allow for comparison to historical conditions so that the results would be applicable for interpreting the existing PET literature. For that purpose, data were acquired using the PET system ECAT EXACT HR, which has a resolution comparable to the currently widely used whole-body PET systems. In relation to the major aims of the study, the results show that automated ROIs generally produce lower BP_ND_ values than manual ROIs, whereas absolute variability was similar. Moreover, the use of realignment improved the absolute variability, and an automated cerebellum ROI yielded slightly increased BP_ND_ values.

The volumes were significantly larger for the automated ROIs, with lower interindividual variability except for the cerebellum, which had larger variability for the automated ROI (Table 2). The differences in volumes between the methods were largest for the caudate and putamen. Similar results of smaller manual compared to FreeSurfer striatal ROIs, but with greater interindividual variability, have recently been reported when analyzing MRI data for the caudate and putamen [22, 51]. Other studies have demonstrated that FS can produce measurements that are comparable to those derived from manual tracing of ROIs [52, 53]. However, manual editing of FS ROIs may still be required in order to improve validity [54].

The intersection of the manual and FreeSurfer ROIs was smaller than the manual ROI itself, which indicates that the ROIs did not overlap completely or in a symmetrical fashion (Table 1). Hence, to shrink or erode the FreeSurfer ROI, as shown for partial volume correction [55], to the same volume as the manual would still result in a placement mismatch.

FreeSurfer has become the standard for obtaining cortical metrics with demonstrated high validity and reliability [56]. For the subcortical regions, manual segmentation still remains the gold standard due to a better specificity of anatomic boundaries compared to FreeSurfer which yields larger caudate and putamen and to FSL-FIRST generating larger caudate ROIs [51, 57]. However, manual subcortical ROI volumes do also vary between different raters [57]. Hence, the differences in the size and placement of the ROIs in the present study may be due to both systematic differences in anatomical designation and in software-based segmentation.

The higher BP_ND_ for the manual ROIs is consistent with their smaller volumes (Table 2). All regions examined have a higher BP when compared with surrounding tissue or the subarachnoidal space. More spill-out than spill-in of the measured radioactivity will yield a recovery coefficient < 1. It is thus conceivable that recovery and BP_ND_ will be lower for a larger delineation of a particular ROI.

After realignment of the dynamic PET images, the BP_ND_ values were significantly higher for the caudate and the dorsolateral prefrontal cortex. For putamen and insula, there was no evident effect of realignment on the BP_ND_ values. A possible explanation for the regional differences in the impact of realignment is that the caudate and dorsolateral prefrontal cortex border to the subarachnoidal or ventricular space where radioactivity is very low or negligible. These regions are thus more sensitive to movement artifacts, while the putamen and insula are embedded in the white matter and subsequently less sensitive.

The use of the modified FSL ROIs for CBL increased the BP_ND_ values by a few percents and decreased the corresponding CoVs for all regions compared to those obtained using the manual cerebellum (Table 2). A possible explanation is that the trimmed and masked FSL ROIs for CBL are less sensitive to the partial volume effects. However, the small difference and narrow CI indicate similarity between these measures and hence the usefulness of both the manual and automated cerebellum ROI.

Generally, the absolute variability decreased significantly after realignment, likely due to the influence of more reliable TACs on the subsequent calculation of the BP_ND_. For the CAU and PUT, which were the regions showing the highest degree of anatomical overlap in our study and therefore represents the best points of comparison, the absolute variability was 6.47 and 3.54% respectively for the automated ROIs (Additional file 1: Table S1). These observations are in line with two previous test-retest studies using manual ROIs to quantify [^11^C]SCH23390 binding. Hirvonen and coworkers investigated five healthy volunteers and found an absolute variability of 4.2–6.6% [58], whereas the absolute variability was 7.8–8.4% in a recent study on 13 individuals [59]. Importantly, the present study shows that the use of automated ROIs has a similar absolute variability of D_1_-DR BP_ND_ values as compared to that of manual ROIs.

The present level of absolute variability among all the brain regions is comparable to that of other PET neuroreceptor radioligands such as [^11^C]raclopride [21, 60], [^11^C]FLB 457 [61], [^11^C]MADAM [62], and [^11^C]AZ10419369 [63]. The repeatability of PET-measured BP_ND_ is of importance to increase the statistical power in receptor occupancy studies, as well as in both cross-sectional and longitudinal studies. For instance, a power analysis based on the present results gives a statistical power to detect a 20% change in BP_ND_ (*n* = 10) in the putamen of 100 and 96% for the manual and FreeSurfer methods, respectively. A 20% change represents a Cohen’s *d* of over 6, which is extremely high for biological changes but commonly seen in drug receptor occupancy studies.

Whereas significant ventricular enlargement and cerebral cortical atrophy are a well-replicated observation in schizophrenia, these changes are usually small and require large sample sizes for detection [64, 65]. Hence, the present findings in healthy volunteers should be applicable in schizophrenia research. Importantly, our results do not support a role for different ROI delineation methods as an explanation for discrepant results in studies on D_1_-R in drug-naïve schizophrenia. In contrast, the present increase in BP_ND_ in putamen and DLPFC with improved absolute variability after realignment may be more relevant. In studies in drug-naïve samples, Okubo et al. found lower D_1_-R in the frontal cortex in patients, whereas Abi-Dargham and colleagues found higher levels [5, 6] and Karlsson et al. found no significant differences, although D_1_-R was numerically higher with an effect size of 0.3 in the frontal cortex [8, 13]. Across all of these papers, movement correction is only described in the 2002 study by Abi-Dargham et al. [5], but no information is provided in the 2012 paper [6]. Although both our previous and the present studies employed head fixation to minimize head movement, the present results show that the absence of movement correction may still produce both lower BP_ND_ and lower reliability. It should be noted that the magnitude of change in BP_ND_ values following movement correction would not be sufficiently large to explain the differences in the direction of the reported clinical findings. However, it may be speculated that movement artifacts may have occurred more commonly among patients than healthy volunteers and subsequently biased the results.

In contrast to the subtle structural brain changes in schizophrenia, gross changes are consistently seen in neurodegenerative diseases such as Alzheimer’s, Parkinson’s, and Huntington’s diseases [66–68]. Structural brain changes may be biased differently by the manual and automated methods, as shown in a recent cross-validation study among automated and manual ROI methods on patients with multiple sclerosis where differences in atrophy measurements both in size and proportionality were demonstrated [69]. Although FreeSurfer has functionality for manual intervention to accommodate for certain morphological changes [54], the present results of similar absolute variability of manual and automated FresSurfer ROI definition (for PET) in healthy subjects need to be confirmed in patient with neurodegenerative disorders.

There are two major advantages of automated ROI definition. First, it is less labor-intensive than tedious hand tracing of ROIs, especially in large high-resolution data sets, and second, it is less biased to rater subjectivity when compared to manual methods. Additionally, automated ROI definition is more amenable for pooling data for meta-analysis, an important aspect since PET studies in patient populations usually have small sample sizes.

## Conclusions

In summary, the results show that the repeatability of BP_ND_ was similar between the manual and FS ROIs, while it improved after realignment in all regions. The results suggest that the choice of the ROI method is more dependent on questions of validity, such as anatomical precision, which may be particularly important in patients with gross morphological changes or very localized pathology. The importance of the correction of motion artifacts by realignment to obtain higher and more reliable BP_ND_ values may be particularly important in patient populations such as schizophrenia where motion artifacts may be more prevalent.

## Additional file

Additional file 1: **Table S1.** Mean BP_ND_ values of [^11^C]SCH23390 in the morning and afternoon, the absolute variability, and ICC in 15 healthy men in four brain regions. There are eight BP_ND_ values for each region derived from the different combinations of methods in the analysis process. (DOCX 18 kb)
