# Supplementary material for: [11C]SCH23390 binding to the D1-dopamine receptor in the human brain—a comparison of manual and automated methods for image analysis
Source: EJNMMI Res. 2018 Aug 2;8:74. doi: 10.1186/s13550-018-0416-2 (PMC6070454; doi:10.1186/s13550-018-0416-2)
Supplement: Supplementary file 1 — Table S1. Mean BPND values of [11C]SCH23390 in the morning and afternoon, the absolute variability, and ICC in 15 healthy men in four brain regions. There are eight BPND values for each region derived from the different combinations of methods in the analysis process. (DOCX 18 kb) [file 13550_2018_416_MOESM1_ESM.docx]

| **ROI** | **Realign** | **CBL** | **ROI Method** | **BP_ND_ Test** | **BP_ND_ Retest** | **AV** | **ICC (CI 95%)** |
| --- | --- | --- | --- | --- | --- | --- | --- |
| **CAU** | **No realign** | **Man CBL** | **Man** | 1.49 | 1.54 | 14.57 | 0.74 (0.39,0.90) |
|  |  |  | **FS** | 1.22 | 1.26 | 14.89 | 0.83 (0.57,0.94) |
|  |  | **Aut CBL** | **Man** | 1.54 | 1.60 | 13.04 | 0.76 (0.43,0.91) |
|  |  |  | **FS** | 1.26 | 1.31 | 13.35 | 0.84 (0.60,0.94) |
|  | **Realign** | **Man CBL** | **Man** | 1.61 | 1.63 | 7.73 | 0.79 (0.48,0.92) |
|  |  |  | **FS** | 1.35 | 1.35 | 8.32 | 0.88 (0.67,0.96) |
|  |  | **Aut CBL** | **Man** | 1.65 | 1.68 | 5.91 | 0.83 (0.58,0.94) |
|  |  |  | **FS** | 1.38 | 1.40 | 6.47 | 0.90 (0.73,0.96) |
| **PUT** | **No realign** | **Man CBL** | **Man** | 1.75 | 1.80 | 6.61 | 0.68 (0.29,0.88) |
|  |  |  | **FS** | 1.55 | 1.59 | 6.41 | 0.78 (0.48,0.92) |
|  |  | **Aut CBL** | **Man** | 1.80 | 1.86 | 5.84 | 0.68 (0.22,0.88) |
|  |  |  | **FS** | 1.60 | 1.65 | 5.46 | 0.79 (0.44,0.92) |
|  | **Realign** | **Man CBL** | **Man** | 1.77 | 1.79 | 4.27 | 0.73 (0.38,0.90) |
|  |  |  | **FS** | 1.57 | 1.58 | 4.22 | 0.84 (0.59,0.94) |
|  |  | **Aut CBL** | **Man** | 1.80 | 1.83 | 3.73 | 0.71 (0.34,0.89) |
|  |  |  | **FS** | 1.61 | 1.63 | 3.54 | 0.83 (0.58,0.94) |
| **DLPC** | **No realign** | **Man CBL** | **Man** | 0.27 | 0.28 | 19.81 | 0.61 (0.17,0.85) |
|  |  |  | **FS** | 0.21 | 0.22 | 25.63 | 0.60 (0.13,0.85) |
|  |  | **Aut CBL** | **Man** | 0.29 | 0.31 | 16.95 | 0.64 (0.23,0.86) |
|  |  |  | **FS** | 0.23 | 0.24 | 19.76 | 0.64 (0.21,0.86) |
|  | **Realign** | **Man CBL** | **Man** | 0.30 | 0.31 | 12.00 | 0.71 (0.33,0.89) |
|  |  |  | **FS** | 0.26 | 0.27 | 13.51 | 0.71 (0.32,0.89) |
|  |  | **Aut CBL** | **Man** | 0.32 | 0.33 | 9.68 | 0.76 (0.43,0.91) |
|  |  |  | **FS** | 0.28 | 0.28 | 9.52 | 0.77 (0.45,0.91) |
| **INS** | **No realign** | **Man CBL** | **Man** | 0.52 | 0.53 | 12.68 | 0.19 (−0.38,0.63) |
|  |  |  | **FS** | 0.52 | 0.51 | 10.78 | 0.28 (−0.29,0.69) |
|  |  | **Aut CBL** | **Man** | 0.54 | 0.56 | 9.63 | 0.44 (−0.06,0.77) |
|  |  |  | **FS** | 0.54 | 0.54 | 6.64 | 0.60 (0.14,0.85) |
|  | **Realign** | **Man CBL** | **Man** | 0.53 | 0.53 | 10.20 | 0.21 (−0.36,0.65) |
|  |  |  | **FS** | 0.52 | 0.51 | 9.41 | 0.35 (−0.20,0.72) |
|  |  | **Aut CBL** | **Man** | 0.55 | 0.56 | 8.92 | 0.40 (−0.14,0.75) |
|  |  |  | **FS** | 0.54 | 0.53 | 6.99 | 0.57 (0.08,0.83) |

Supplementary Table 1. Mean BP_ND_ values of [^11^C]SCH23390 in the morning and afternoon, the absolute variability and ICC in 15 healthy men in four brain regions. There are eight BP_ND_ values for each region derived from the different combinations of methods in the analysis process.
